# Extension Educators and Volunteer Leaders: Evaluation of Fitness Outcomes Among Participants in Community Strength Training Classes

**DOI:** 10.3389/fpubh.2020.566387

**Published:** 2020-10-19

**Authors:** Lisa Tucker Washburn, LaVona Traywick, M. E. Betsy Garrison

**Affiliations:** ^1^Department of Family and Consumer Sciences, University of Tennessee Institute of Agriculture, Knoxville, TN, United States; ^2^Arkansas Colleges of Health Education, Fort Smith, AR, United States; ^3^School of Human Environmental Sciences, University of Arkansas, Fayetteville, AR, United States

**Keywords:** strength training, community-based, Cooperative Extension, volunteer leaders, older adults, rural outreach, fitness outcomes, lay leaders

## Abstract

Volunteer-led strength training classes can expand access, improve exercise adherence, and enhance intervention sustainability for older adults. This study compared participant functional fitness outcomes between volunteer-led and Extension educator-led StrongWomen strength training groups in community settings. Change scores for participants (*n* = 317) were calculated for six Senior Fitness Test (SFT) measures. A non-parametric analysis of independent samples to determine SFT score differences between participant groups (educator-led and volunteer-led) showed no significant differences. Volunteers and professionals, like Extension educators, may be similarly effective in conducting community-based strength training classes resulting in improved functional fitness outcomes. We offer recommendations for organizations seeking to adopt similar approaches.

## Introduction

As Americans age, their participation in muscle strengthening exercise declines. According to one recent study, strength training participation begins to fall around midlife. Prevalence is lowest among those age 75 and older, when only 21% meet recommendations of twice weekly participation (1). Another study comparing the proportion of older adults meeting strength training recommendations across three large, national studies reported prevalence ranging from 16.7 to 21.6% (2).

Regular strength training is associated with reduced fall risk, reduced disability, and improved chronic disease management, all conditions disproportionately affecting older adults (3–7). Given the well-established and wide ranging benefits of strength training, low prevalence among older adults is a valid concern.

Older adult's exercise barriers include time commitments, geographic proximity to exercise facilities, transportation, and lack of social support (8–11). Older adults in rural areas face additional barriers related to geographic isolation and limited financial resources for fee-based strength training classes or facilities (12, 13). New approaches are needed to reduce barriers and increase the proportion of older adults regularly engaging in strengthening activities. Peer or lay leadership of community-based programs is one approach with promise for increasing strength training and improving functional fitness outcomes among older adults (14).

Peer and lay leaders have become common elements of community-based intervention efforts, but no generally agreed upon definition for these terms exists (15, 16). The terms “peer,” “volunteer,” and “lay” are often used interchangeably in the literature, although important differences may exist depending on intervention type and implementation setting. Lay leaders, who may or may not be peers, have been referred to as lay health advisors, community health workers, community health aides, and community health advisors in health promotion literature (17). These leaders may be similar to the target audience in age, income level, racial or ethnic group, or residential geography. Lay leaders are typically nonprofessional community members trained to deliver specific interventions (18, 19).

## Literature Review

Strategies to increase older adults' physical activity levels have been the focus of numerous studies (20–22). Benefits of strength training for older adults are well-established (23). Structured community-based programs are an effective strategy (6, 7, 24–26). For example, study of fitness outcomes in the StrongWomen Program (SWP), a nationally-disseminated and community-based strength training program, showed older adult women significantly improved functional fitness in six domains assessed by the Senior Fitness Test (SFT) (27–29). The SFT is a criterion-referenced test based on scores for thousands of older adults. Other programs using selected domains of the SFT to evaluate participant outcomes in community-based settings, such as EnhanceFitness and Stay Strong, Stay Healthy (SSSH), found improved functional fitness (30, 31). Fitness improvements in SWP, EnhanceFitness, and SSSH were attained by instructors with and without specialized exercise backgrounds. The SWP and SSSH programs are typically led by non-exercise professionals trained to instruct the structured classes. EnhanceFitness was originally instructed by certified fitness trainers after receiving additional, program-specific training (30).

Published studies exploring use of volunteers or lay leaders in health outreach programs, including group exercise and strength training classes, have increased in the last decade (24, 32–37). A recent review of peer-led healthy aging programs implemented in practical settings found only 12 published studies suitable for inclusion. Of six peer-led programs included, three involved peer leaders in strength training instruction (14). While more practice-based evidence is needed supporting adoption of peer-led strategies to increase older adult strength training, including lay persons in program delivery clearly offers benefits for program participants (19, 38). Additionally, increased access afforded through peer-led programs has potential to positively impact public health (38, 39).

At the individual level, similar participant outcomes have been found in volunteer-led and professionally-led programs addressing a range of health conditions. Outcomes for volunteer-led groups were found comparable for arthritis self-management, falls prevention, chronic disease self-management, and physical activity (33, 40, 41). From a relational perspective, volunteer leaders may be more effective than professionals and can serve as powerful role models (42). Advice and new knowledge may be more readily accepted and digested in an atmosphere of shared common experiences (43). Volunteer-led approaches also promote self-efficacy, a major determinant of physical activity maintenance (44). Volunteer leaders can advise participants of potential challenges in being physically active, communicate strategies to deal with them, and increase participant self-efficacy in dealing with difficulties (45). Self-efficacy is important in exercise adoption and can decrease attrition from exercise programs (46, 47). Additionally, volunteer-led group strength training classes can improve exercise adherence and maintenance (19, 48).

Improving access to strength and balance programming is an important public health strategy; however, access is limited when programs rely on professionals for instruction (e.g., physical therapists, nurses), particularly when teaching exercise classes draws professionals away from clinical activities (37, 49). A recent systematic review found that availability of organized exercise opportunities was the most common environmental factor motivating older adults' strength training engagement. Access to facilities was a commonly cited motivator (11); lack of access has been cited as a barrier (12). A majority of community-based group fitness classes are led by health or fitness professionals, or others with certifications or degrees in Kinesiology and related fields (6, 19). One exception is the open-access community-based physical activity programs offered by Extension educators operating through the Land-Grant University Cooperative Extension System (21). Extension educators, who typically have bachelor's or master's degrees in areas unrelated to exercise science, offer programs through a network of county Extension offices in each state and are supported by campus-based faculty in their respective states.

Program models including volunteer leaders is one strategy to address access barriers of cost and transportation, particularly for rural older adults who typically have limited access to evidence-based programs (39). Capacity for implementation limits program dissemination to high-need areas and access by populations who stand to benefit most. Lay volunteers, as nonprofessional community members, are an important complement to professional healthcare providers given the shortage of people currently qualified to address physical activity needs of the growing older adult population (15, 16, 18, 37, 50).

Volunteer leadership of programs may enhance sustainability and reach (24, 39, 51, 52). Community-based program sustainability is important to make long-term differences in health behaviors, which is vital for rural older adults who are less healthy and more sedentary than their urban counterparts (53, 54). One study found counties using volunteer leaders to instruct strength training groups were 8.3 times more likely to have continuing groups compared with counties not using volunteer leaders (51).

Some studies have compared volunteer-led groups and a control group, but few reported functional fitness outcomes and drew comparisons between volunteer-led and professionally-led approaches in real-world, community-based settings (26, 33, 35, 52). The study described here examined fitness improvements among participants of two types: those in counties adopting a volunteer-led program model, and those relying solely on an educator-led model for StrongWomen Program (SWP) delivery. This article provides a brief description of the SWP and the implementation approach involving Extension educators and volunteers in one state. Then, findings from analysis of functional fitness outcomes among participants, and comparison of participant improvements in counties adopting the volunteer-led or educator-led model, are described.

Here, “volunteer leader” is used in reference to unpaid laypersons trained to teach structured community-based strength training classes. In this context, volunteer leaders usually do not have specialized fitness certifications but have received training to lead a specific sequence of exercises under supervision of a community education professional employed by an organization. For clarity, the terms “lay” and “peer” are used when referring to studies using this terminology when volunteer status was not reported.

## Program Approach

The SWP is an evidence-based strength training program for mid-life and older women. The SWP was designed to be community-based and implemented through non-profit organizations and settings by trained SWP leaders (55). The program consists of hour-long strength training sessions held twice weekly over 12 weeks. Individual sessions include a warm-up, 8–10 strengthening exercises using dumbbells and ankle weights, and a cool-down and stretch. Classes met in various sites, most commonly community centers, churches, and meeting rooms located in county Extension offices.

Nationally, the SWP is most widely implemented through the Cooperative Extension Service (Extension). Extension programs are delivered by professional Extension educators housed in county-based offices. Extension educators are paid employees, typically non-exercise professionals, hold a bachelor's or master's degree, and conduct programming in a variety of areas. Extension educators implementing the SWP in Arkansas had a range of educational backgrounds, including family and consumer sciences, nutrition, and health education. Extension educators implement programs based on local needs, which usually includes some aspect of health and wellness.

Extension educators leading the SWP completed the StrongWomen Program Leader Workshop, where they received the StrongWomen Toolkit developed by the Tufts University StrongWomen team to guide implementation (56). Workshops in Arkansas were conducted by a StrongWomen Ambassador team comprised of Extension educators in the state who were long-term program leaders with extensive training and SWP mentorship. The strength training program, including training for Program Leaders (those trained to lead local classes, regardless of volunteer or professional status) and the StrongWomen Tool Kit, have been described elsewhere (27, 55).

The SWP in Arkansas evolved to include instruction by volunteers recruited from an initial SWP class led by the county Extension educator (51), but this did not occur in every county. In counties where volunteer instructors were not recruited, classes were solely led by the Extension educator. Consistent with SWP protocols, both Extension educators and volunteer leaders were trained by StrongWomen Ambassadors using a standardized format and materials (55).

In counties utilizing the volunteer delivery model, the Extension educator remained responsible for program oversight, including evaluation, maintaining paperwork, and conducting site visits to monitor progress and ensure program fidelity. In both class formats—educator-led or volunteer-led—the Extension educator conducted the SFT to evaluate participant functional fitness improvements (28).

## Methods

### Participants

The initial sample consisted of 658 SWP participants in volunteer- and educator-led classes who had completed a baseline SFT at or near the beginning of the program. Of these, 317 participants completed a subsequent SFT at or near 12 weeks after program start and were included in the analysis. Participants were categorized as volunteer-led or educator-led based on county of SWP class attendance. Counties designated educator-led did not have one or more trained SWP leaders aside from the Extension agent. The presence of one or more volunteers trained as SWP leaders determined county classification as volunteer-led. Participants represented 22 counties with ages ranging from 27 to 95 years old (*M* = 66.51; *SD* = 12.00) and BMIs ranging from 18 to 51 (*M* = 29.00; *SD* = 6.49). Almost all participants were White women living in a rural areas. Nearly three-quarters of participants (*n* = 234) were in volunteer-led counties. The Institutional Review Board of the affiliated university approved the procedures used in this study.

### Setting

The SWP targets mid-life and older women, but was open to adults of all ages; a majority of participants were female older adults. SWP participants included in the sample attended strength training classes led by the county Extension educator (educator-led), or were in counties utilizing volunteer leaders to instruct programs (volunteer-led). Participants in counties classified as volunteer-led were instructed by volunteers all or some of the SWP sessions. The program delivery model used in this state recommended new SWP classes be initiated and led by the Extension agent for the first 12 weeks, but classes frequently transitioned to volunteer leadership before the initial 12 week period concluded. Volunteer leaders enabled ongoing classes which continued indefinitely and beyond the initial 12 weeks. New participants could join existing SWP classes at any time. Participants attending classes in educator-led counties were led by the county Extension educator. Participants in volunteer-led counties may have attended classes taught solely by volunteers or taught by both the educator and volunteer leaders. Regardless of leader type, all sessions were conducted by instructors completing the standardized SWP Leader Workshop and complied with program protocols.

### Data Collection

Extension educators were trained and conducted participant SFT using standardized protocols. Data were reported to a web-based data management system and exported for purposes of the analysis described here. The SFT is criterion-referenced by age and gender and was selected as most appropriate for the project given participants' age range, the need for measure consistency across participant groups, and ease of administration. The SFT consists of six functional fitness measures, plus height and weight to determine body mass index (BMI). Focus area and aligning measures include: upper body flexibility: back-scratch test; lower body flexibility: sit-and-reach test; agility and dynamic balance: 8-foot up-and-go test; upper body strength: arm curl test; lower body strength: chair stand test; aerobic endurance: 3-min step test (28).

### Data Analysis

Baseline SFT data and 12-week follow-up data were matched for participants (*n* = 317). To account for differences in individuals, six change scores, one for each SFT measure excluding BMI, were calculated by subtracting the post-scores from the initial scores. Following frequency analysis and a determination that not all variables were normally distributed, a non-parametric analysis of independent samples with a Mann–Whitney *U*-test statistic was performed to determine any statistically significant differences on the SFT change scores between group types. Data were analyzed using SPSS version 26 (IBM, Chicago).

## Results

Regardless of county classification by leader type (educator- or volunteer-led), a majority of participants improved on all measures of functional fitness, except agility and dynamic balance. Sixty-six percent of participants (*n* = 209) improved lower body strength as indicated by improved chair stand test scores; 64% (*n* = 204) improved upper body strength measured by the arm curl test. Improvements in aerobic endurance were observed among 66% of participants (*n* = 209). Lower body flexibility, measured by the chair sit-and-reach test, was improved by 57% of participants (*n* = 180). A slight majority of participants, 51% (*n* = 161), improved upper body flexibility. Agility and dynamic balance, measured by the 8-foot up-and-go test, were improved by 42% (*n* = 133) of participants.

Table 1 lists change score means and standard deviation by county leader type, *U*-scores, and significance level for each functional fitness variable. There were no statistically significant differences between the educator-led and volunteer-led participant groups. Community-based strength training classes led by volunteer leaders and Extension educators may obtain similar improvements in functional fitness outcomes.

**Table 1:** Comparison of change scores for six functional fitness measures by leader type.

		**Volunteer-led**		**Educator-led**			
**Measure**	***n***	**Mean**	**SD**	**Mean**	**SD**	***U***	***p***
Lower Body Strength	308	1.79	4.17	1.94	4.40	9,446	0.794
Upper Body Strength	314	2.18	6.04	1.98	5.81	9,559	0.887
Endurance	311	10.90	29.66	7.60	25.54	−8,689	0.316
Lower Body Flexibility	309	0.69	3.51	0.99	3.30	9,391	0.903
Upper Body Flexibility	312	0.09	5.13	−0.99	3.56	8,459	0.137
Agility and Dynamic Balance	314	0.38	1.41	0.60	1.80	9,832	0.79

## Discussion

Findings of no significant difference in fitness outcomes among participants in volunteer-led and Extension educator-led counties lends support to the value of volunteer approaches in community-based programs. Concerns about volunteer leader effectiveness and program quality have been voiced as rationale for not adopting this approach. These findings help address such concerns and support adoption and implementation of outreach strategies with intentional involvement of volunteer leaders. Implications for program design, implementation, and sustainability extend beyond the niche of group exercise classes. Designing programs with intent for delivery by volunteer leaders, given that similar participant outcomes are expected as with professional leaders, can help ensure program continuance and expansion following the initial implementation period. Based on findings, we can now offer more definitively recommended strategies for organizations aiming to develop or adapt volunteer-led health programs.

### Program Planning

Health outreach programs planned with intent to engage volunteers from the outset will be more easily implemented (57). Program structure, as key to successful volunteer leader engagement, should explicitly identify volunteer leader roles. Volunteer leaders may offer high value and return on investment for organizations and should be engaged in stimulating roles providing opportunities to practice new skills or build on existing personal strengths (58, 59).

### Volunteer Leader Recruitment

Active volunteer leader recruitment strategies, including personal invitations to potential volunteers, are recommended over passive methods (e.g., newspaper articles/ads, fliers, brochures), which require interested individuals to initiate action (60, 61). However, program implementers should be careful to avoid over-recruiting from the same pool of volunteers from convenience or familiarity. Research in other volunteer-led programs found reframing ideas about who volunteers and where volunteers can be found is essential for program growth (62).

### Volunteer Leader Training

Proper training is imperative to ensure program fidelity and volunteer leader success (16). In the program described here, volunteer leaders and Extension educators received standardized face-to-face training on the evidence-based program protocol (56). Volunteers' effectiveness in achieving similar participant fitness outcomes as professionals suggests volunteer leaders should be trained using the same methods and level of rigor often reserved for professionals. Face-to-face training was necessary to adhere to the SWP Leader Training protocol and allowed trainers to evaluate both volunteer leaders and Extension educators form and technique in teaching strengthening exercises. The initial time and monetary investment in training was intensive and may have been reduced by utilizing other methods, such as web-based training. However, this training modality may be inappropriate for initial training of group exercise leaders, even when local classes are supervised by the Extension educator to ensure program adherence. The trend toward offering more web-based training may be suitable for some community-based programs, but effectiveness for exercise classes needs further evaluation. Program planners should carefully weigh costs and benefits of face-to-face vs. indirect training methods to determine the best strategy (63).

### Volunteer Leader Engagement and Motivation

Understanding factors motivating volunteer engagement and continued involvement is key. Motivational factors include achievement, affiliation, and power and can be extrinsic or intrinsic. Motivators for initiating service may differ from motivators for continuing service (64). Learning new things, helping others, and a sense of obligation to the community are intrinsic motivators for volunteers. Motivational strategies for organizations include continued training, which provides opportunities for growth and development; creating opportunities for volunteer leaders to give and receive social support; and formal and informal recognition (37).

Recommendations for future studies include replicating this study to demonstrate volunteer leader efficacy as well as consideration of leader and program participant demographic characteristics. Additionally, future studies should explicitly investigate effectiveness of leader training format—face-to-face, web-based, or a hybrid approach combining both. Lastly, future studies should examine adoption of similar volunteer-led approaches for programs originally developed for professional delivery.

### Limitations

Personal and professional backgrounds of the volunteer leaders in this study, including educational status, is unknown. Prior research on perceived factors supporting volunteer leader model adoption among implementing Extension educators found volunteer leaders' education level to be an influential factor (65). A multi-state study found trained leaders with a bachelor's degree or higher were more likely to implement the SWP than those with lower education levels (66). In this single state study, volunteer leaders with professional work experience, such as retired teachers, or past athletic participation, may have been more likely to volunteer than others which could influence their success in this type of role. However, the influence of such factors cannot be accounted for here.

The absence of participant characteristics from our analyses, such as education, age, race and ethnicity, professional background, and economic status is an additional limitation potentially impacting participant outcomes. Aside from age and gender, these data were not collected as part of the SFT or SWP evaluation protocol. A further limitation is the proportion of the initial sample included in data analyses. Of 658 participants completing baseline SFT at or near the start of the SWP, 48% (*n* = 317) were eligible for inclusion. Participants with subsequent SFT delayed beyond 12 weeks were excluded to ensure consistency in comparisons.

This analysis utilized secondary data from a community-based program in 22 counties. Baseline and follow-up assessments were matched at the participant level with county of class attendance identified. However, leader type was not built into program reporting protocols and direct comparisons between classes led by volunteers and those led by Extension educators are not possible. Further, some instructor crossover is likely, as groups could have been led by both Extension educators and volunteers during the 12-week period. The aim of this study was not to establish volunteer or educator-led classes as “better,” but to explore whether fitness outcomes were comparable between participants in counties using either delivery models. Future study exploring differences in participant fitness outcomes by class delivery method and leader characteristics would further contribute to the literature.

Although the SWP targets mid-life and older women, programs offered by Extension are open access and cannot be limited, by law, to specific gender and age groups. Program protocols include use of the SFT as a functional fitness measure, although established ranges of functional ability are not available for those younger than 60 years of age. Use of the SFT is a potential limitation, given the age range of participants in the sample, but is addressed by data analysis methods. Change scores were calculated by comparing individual participants' 12-week follow-up assessment to baseline assessment data and was unrelated to the age and gender ranges established in the SFT.

Several issues made use of a control or comparison group unfeasible. Secondary data produced from real-world implementation of a community-based program were analyzed, which did not allow for experimental or quasi-experimental design. Despite this limitation, the analysis presented is among the first to explore differences in participant fitness outcomes in counties with volunteer leadership and counties with Extension educator leadership, contributing to a gap in the literature regarding effectiveness of volunteer-delivered strength training programs.

## Conclusion

Demand for community-based fitness programs will remain unmet without adoption of new approaches to expand reach (16, 24). The shortage of programs, combined with limited time available for professionals to offer such programs, points to the promise and importance of using volunteer leaders to bridge the gap. Adaptation of evidence-based programs for instruction by volunteer leaders offers an alternative to professional instruction and a feasible approach to increase reach.

The volunteer leaders and Extension educators in this study were equally effective in teaching the exercise program, as evidenced by improved functional fitness among participants. The finding of no significance between group functional fitness outcomes is likely due to use of a standardized protocol for training and adaptation of a research-based strength training program for implementation in community settings. Organizations seeking to implement similar programs should be mindful of training needs and capacity of lay leaders or volunteers. Training and support provided should be high-quality and equally as rigorous as training provided to professionals delivering the same program.

## Data Availability Statement

Requests to access these datasets should be directed to M.E. Betsy Garrison, megarris@uark.edu.

## Ethics Statement

The studies involving human participants were reviewed and approved by University of Arkansas Institutional Review Board. Written informed consent for participation was not required for this study in accordance with the national legislation and the institutional requirements.

## Author Contributions

All authors jointly developed study design. LW and LT coordinated data collection. LW led manuscript preparation. MG conducted data analysis and interpretation. All authors read, contributed to, and approved the final manuscript.

## Conflict of Interest

The authors declare that the research was conducted in the absence of any commercial or financial relationships that could be construed as a potential conflict of interest.

## References

[1] Bennie JA Lee DC Khan A Wiesner GH Bauman AE Stamatakis E. Muscle-strengthening exercise among 397,423 U.S. adults: prevalence, correlates, and associations with health conditions. Am J Prev Med. (2018) 55:864–74. 10.1016/j.amepre.2018.07.022 30458949

[2] Keadle S McKinnon R Graubard B Troiano R. Prevalence and trends in physical activity among older adults in the United States: a comparison across three national surveys. Prev Med. (2016) 89:37–43. 10.1016/j.ypmed.2016.05.009 27196146 PMC4969157

[3] Braith R Stewart K. Resistance exercise training: its role in the prevention of cardiovascular disease. Circulation. (2006) 113:2642–50. 10.1161/CIRCULATIONAHA.105.584060 16754812

[4] Chevan J. Demographic determinants of participation in strength training activities among U.S. adults. J Strength Cond Res. (2008) 22:553–58. 10.1519/JSC.0b013e3181636bee 18550973

[5] Williams MA Haskell WL Ades PA Amsterdam EA Bittner V Franklin BA. Resistance exercise in individuals with and without cardiovascular disease: 2007 update: a scientific statement from the American Heart Association Council on Clinical Cardiology and Council on Nutrition, Physical Activity, and Metabolism. Circulation. (2007) 116:572–84. 10.1161/CIRCULATIONAHA.107.185214 17638929

[6] Waters DL Hale LA Robertson L Hale BA Herbison P. Evaluation of a peer-led falls prevention program for older adults. Arch Phys Med Rehabil. (2011) 92:1581–6. 10.1016/j.apmr.2011.05.014 21963125

[7] Wurzer B Waters DL Hale LA Leon de la Barra S. Long-term participation in peer-led fall prevention classes predicts lower fall incidence. Arch Phys Med Rehabil. (2014) 95:1060–6. 10.1016/j.apmr.2014.01.018 24508186

[8] Lees F Clark P Nigg C Newman P. Barriers to exercise behavior among older adults: a focus-group study. J Aging Phys Act. (2005) 13:23–33. 10.1123/japa.13.1.23 15677833

[9] Cleary K LaPier T Rippee A. Perceptions of exercise and quality of life in older patients in the United States during the first year following coronary artery bypass surgery. Physiother Theory Pract. (2015) 31:337–46. 10.3109/09593985.2015.1004770 25630389

[10] Arcury T Preisser J Gesler W Powers J. Access to transportation and health care utilization in a rural region. J Rural Health. (2005) 21:31–8. 10.1111/j.1748-0361.2005.tb00059.x 15667007

[11] Burton E Farrier K Lewin G Pettigrew S Hill A Airey P. Motivators and barriers for older people participating in resistance training: a systematic review. J Aging Phys Act. (2017) 25:311–24. 10.1123/japa.2015-0289 27620535

[12] Korkiakangas EE Alahuhta MA Laitinen JH Barriers to regular exercise among adults at high risk or diagnosed with type 2 diabetes: a systematic review. Health Promot Int. (2009) 24:416–27. 10.1093/heapro/dap031 19793763

[13] Nied RJ Franklin B. Promoting and prescribing exercise for the elderly. Am Fam Physician. (2002) 65:419–26. 11858624

[14] Wurzer BM Hurkmans EJ Waters DL The use of peer-led community-based programs to promote healthy aging. Curr Geriatr Rep. (2017) 6:202–11. 10.1007/s13670-017-0217-x

[15] Webel AR Okonsky J Trompeta J Holzemer WL. A systematic review of the effectiveness of peer-based interventions on health-related behaviors in adults. Am J Public Health. (2010) 100:247–53. 10.2105/AJPH.2008.149419 20019321 PMC2804647

[16] Schneider EC Altpeter M Whitelaw N. An innovative approach for building health promotion program capacity: a generic volunteer training curriculum. Gerontologist. (2007) 47:398–403. 10.1093/geront/47.3.398 17565104

[17] United States Department of Health and Human Services, Health Research and Services Administration, Bureau of Health Professions Community Health Worker National Workforce Study. (2007). Available online at: https://bhw.hrsa.gov/sites/default/files/bhw/nchwa/projections/communityhealthworkforce.pdf

[18] Simoni JM Franks JC Lehavot K Yard SS. Peer interventions to promote health: conceptual considerations. Am J Orthopsychiatry. (2011) 81:351–9. 10.1111/j.1939-0025.2011.01103.x 21729015 PMC3607369

[19] Burton E Farrier K Hill KD Codde J Airey P Hill A Effectiveness of peers in delivering programs or motivating older people to increase their participation in physical activity: systematic review and meta-analysis. J Sports Sci. (2018) 36:666–78. 10.1080/02640414.2017.1329549 28535358

[20] Conn V Minor M Burks K Rantz M Pomeroy S. Integrative review of physical activity intervention research with aging adults. J Am Geriatr Soc. (2003) 51:1159–68. 10.1046/j.1532-5415.2003.51365.x 12890083

[21] Balis LE Strayer T Ramalingam N Wilson M Harden SM. Open-access physical activity programs for older adults: a pragmatic and systematic review. Gerontologist. (2019) 59:e268–78. 10.1093/geront/gnx195 29329395

[22] Devereux-Fitzgerald A Powell R Dewhurst A French DP. The acceptability of physical activity interventions to older adults: a systematic review and meta-synthesis. Soc Sci Med. (2016) 158:14–23. 10.1016/j.socscimed.2016.04.006 27104307

[23] Liu C Latham N. Progressive resistance strength training for improving physical function in older adults (review). Cochrane Database Syst Rev. (2009) 2009:1–212. 10.1002/14651858.CD002759.pub2 19588334 PMC4324332

[24] Werner D Teufel J Brown SL Evaluation of a peer-led, low-intensity physical activity program for older adults. Am J Health Educ. (2014) 45:133–41. 10.1080/19325037.2014.893851

[25] Layne JE Arabelovic S Wilson LB Cloutier GJ Pindrus MA Mallio CJ Community-based strength training improves physical function in older women with arthritis. Am J Lifestyle Med. (2009) 3:466–73. 10.1177/1559827609342061

[26] Yan T Wilber K Aguirre R Trejo L. Do sedentary older adults benefit from community-based exercise? Results from the Active Start Program. Gerontologist. (2009) 49:847–55. 10.1093/geront/gnp113 19592637

[27] Seguin R Kuder J Heidkamp-Young E Nelson M. Improved physical fitness among older female participants in a nationally disseminated, community-based exercise program. Health Educ Behav. (2012) 39:183–90. 10.1177/1090198111426768 22234966 PMC3727397

[28] Rikli R Jones C. Senior Fitness Test Manual. 2nd ed. Champaign, IL: Human Kinetics (2013).

[29] Chaudhary AK Van Horn B Corbin M StrongWomen Program evaluation: effect of strength training exercises on physical fitness of participants. J Ext. (2015) 53.

[30] Belza B Schumway-Cook A Phelan E Williams B Snyder S LoGerfo J The effects of a community-based exercise program on function and health in older adults: the EnhanceFitness Program. J Appl Gerontol. (2006) 25:291–306. 10.1177/0733464806290934

[31] Ball S Gammon R Kelly P Cheng AL Chertoff K Kaume L. Outcomes of Stay Strong, Stay Healthy in community settings. J Aging Health. (2013) 25:1388–97. 10.1177/0898264313507318 24150062

[32] Layne J Sampson S Mallio C Hibberd P Griffith J Krupa Das S. Successful dissemination of a community-based strength training program for older adults by peer and professional leaders: the People Exercising Program. J Am Geriatr Soc. (2008) 56:2323–9. 10.1111/j.1532-5415.2008.02010.x 19112654

[33] Tudor-Locke C Lauzon N Myers A Bell R Chan C McCargar L. Effectiveness of the First Step Program delivered by professionals versus peers. J Phys Act Health. (2009) 6:456–62. 10.1123/jpah.6.4.456 19842459

[34] Nanduri AP Fullman S Morell L Buyske S Wagner ML. Pilot study for implementing an osteoporosis education and exercise program in an assisted living facility and senior community. J Appl Gerontol. (2018) 37:745–62. 10.1177/0733464816672045 27733660

[35] Buman MP Giacobbi PR Jr Dzierzewski JM Aiken Morgan A McCrae CS Roberts BL Peer volunteers improve long-term maintenance of physical activity with older adults: a randomized controlled trial. J Phys Act Health. (2011) 8:S257–66. 10.1123/jpah.8.s2.s257 PMC3181088 21918240

[36] Sciamanna C Ballentine NH Bopp M Brach JS Chinchilli VM Ciccolo JT. Working to Increase Stability through Exercise (WISE): study protocol for a pragmatic randomized controlled trial of a coached exercise program to reduce serious fall-related injuries. Contemp Clin Trials. (2018) 74:1–10. 10.1016/j.cct.2018.09.006 30261294 PMC6333097

[37] Larkin M Maximizing the potential of lay leaders. J Active Aging. (2008) 2008:29–34.

[38] Green LW Glasgow RE. Evaluating the relevance, generalization, and applicability of research: issues in external validation and translation methodology. Eval Health Prof. (2006) 29:126–53. 10.1177/0163278705284445 16510882

[39] Ory MG Smith ML. Research, practice, and policy perspectives on evidence-based programing for older adults. Front Public Health. (2015) 3:1–9. 10.3389/fpubh.2015.00136 25973417 PMC4411719

[40] Lorig K Ritter P Laurent D Fries J. Long-term randomized controlled trial of tailored-print and small-group arthritis self-management interventions. Med Care. (2004) 42:346–54. 10.1097/01.mlr.0000118709.74348.65 15076811

[41] Healy T Peng C Haynes P McMahon E Botler J Gross L The feasibility and effectiveness of translating 'A Matter of Balance' into a volunteer lay leader model. J Appl Gerontol. (2008) 27:34–51. 10.1177/0733464807308620

[42] Hainsworth J Barlow J The training experiences of older, volunteer lay leaders on an arthritis self-management course. Health Educ J. (2003) 62:266–77. 10.1177/001789690306200308

[43] Peel N Warburton J. Using senior volunteers as peer educators: what is the evidence of effectiveness in falls prevention? Australas J Ageing. (2009) 28:7–11. 10.1111/j.1741-6612.2008.00320.x 19243369

[44] Walters C Troutman-Jordan M An investigation of the effectiveness of A Matter of Balance/Volunteer Lay Leader Model (AMOB/VLL): findings from a community senior center. Act Adapt Aging. (2018) 42:69–80. 10.1080/01924788.2017.1376174

[45] McAuley E Jerome G Marquez D Elvasky S Blissmer B. Exercise self-efficacy in older adults: social, affective, and behavioral influences. Ann Behav Med. (2003) 25:1–7. 10.1207/S15324796ABM2501_01 12581930

[46] McAuley E Courneya K Rudolph D Lox C. Ehancing exercise adherence in middle-aged males and females. Prev Med. (1994) 23:498–506. 10.1006/pmed.1994.1068 7971878

[47] Sniehotta FF Scholz U Schwarzer R Bridging the intention-behaviour gap: planning, self-efficacy, and action control in the adoption and maintenance of physical exercise. Psychol Health. (2005) 20:143–60. 10.1080/08870440512331317670

[48] Kim S Koniak-Griffin D Flaskerud J Guarnero P. The impact of lay health advisors on cardiovascular health promotion. J Cardiovasc Nurs. (2004) 19:192–9. 10.1097/00005082-200405000-00008 15191262

[49] Taing D McKay K. Better Strength, Better Balance! Partnering to deliver a fall prevention program for older adults. Can J Public Health. (2017) 108:e314–e9. 10.17269/CJPH.108.5901 28910255 PMC6972297

[50] Studer S von Schnurbein G Organizational factors affecting volunteers: a literature review on volunteer coordination. Voluntas. (2013) 24:403–40. 10.1007/s11266-012-9268-y

[51] Washburn LT Cornell CC Phillips ME Felix H Traywick L. Strength training in community settings: impact of lay leaders on program access and sustainability for rural older adults. J Phys Act Health. (2014) 11:1408–14. 10.1123/jpah.2013-0007 24368673

[52] Izutsu K Arima K Abe Y Okabe T Tomita Y Mizukami S. Exercise intervention implemented by trained volunteers improves health-related quality of life among Japanese community-dwelling older females: an intervention study. J Phys Ther Sci. (2017) 29:2126–32. 10.1589/jpts.29.2126 29643589 PMC5890215

[53] Fan JX Wen M Kowaleski-Jones L. Rural-urban differences in objective and subjective measures of physical activity: findings from the National Health and Nutrition Examination Survey (NHANES) 2003-2006. Prev Chronic Dis. (2014) 11:E141. 10.5888/pcd11.140189 25144676 PMC4149321

[54] Jones C Parker T Ahearn M Mishra AK Variyam J Health Status and Health Care Access of Farm and Rural Populations. United States Department of Agriculture, Economic Research Service (2009) Available online at: https://www.ers.usda.gov/webdocs/publications/44424/9370_eib57_reportsummary_1_.pdf

[55] Seguin R Economos C Hyatt R Palombo R Reed P Nelson M. Design and national dissemination of the StrongWomen community strength training program. Prev Chronic Dis. (2008) 5:A25. 18082014 PMC2248774

[56] Nelson M Seguin R. The StrongWomen Tool Kit. Boston, MA: Tufts University (2005).

[57] Brudney J Designing and Managing Volunteer Programs. In: Renz DO, editor. The Jossey-Bass Handbook of Nonprofit Leadership and Management. 3rd ed. San Francisco: Jossey-Bass (2010). p. 753–93.

[58] Snider A The dynamic tension: professionals and volunteers. J Ext. (1985) 23:7–10.

[59] Washburn L Cornell CE Traywick L Felix HC Phillips M Volunteer delivery of a community-based strength training program: comparison of adopting and nonadopting Extension educator perspectives. J Hum Sci Ext. (2016) 4:124–42.

[60] Farris E McKinley S Ayres J Peters J Brady C County-level extension leadership: understanding volunteer board member motivation. J Ext. (2009) 47.

[61] Washburn LT Cornell CC Traywick L Felix H Phillips ME Motivations of volunteer leaders in an extension exercise program. J Ext. (2015) 53.

[62] Washburn LT Traywick L Copeland L Vincent J Extension Wellness Ambassadors: Individual effects of participation in a health-focused master volunteer program. J Ext. (2017) 55.

[63] Conte KP Held F Pipitone O Bowman S. The feasibility of recruiting and training lay leaders during real-world program delivery and scale-up: the case of Walk With Ease. Health Promot Pract. (2019). 10.1177/1524839919840004. [Epub ahead of print]. 30971154

[64] Wolford M Cox K Culp K Effective motivators for master volunteer program development. J Ext. (2001) 39.

[65] Washburn LT Cornell CE Traywick L Felix HC Phillips ME Barriers and facilitators to adoption of a lay-delivered community-based strength training program for women in rural areas. Am J Health Educ. (2017) 48:156–66. 10.1080/19325037.2017.1292970

[66] Seguin R Palombo R Economos C Hyatt R Kuder J Nelson M. Factors related to leader implementation of a nationally disseminated community-based exercise program: a cross-sectional study. Int J Behav Nutr Phys Act. (2008) 5:62. 10.1186/1479-5868-5-62 19055821 PMC2614422

